# Co-Operation in a Contributory Scheme

**Published:** 1922-12

**Authors:** W. Fenn

**Affiliations:** Chaplain. General Infirmary, Salisbury


### Co-operation in a Contributory Scheme.

*To the Editor of* The Hospital and Health Review.

Sir,—With reference to Dr. Luckham's letter which appeared in your October issue, it has occurred to me that some of your readers might like to read the original form of the scheme which was my contribution to the negotiations which have resulted in the passing of the resolutions recorded by Dr. Luckham. The original draft of my scheme, dated November 30, 1921, in substance is as follows :—

1. It is suggested that a society should be formed and known by some general name—*e.g.*, "The Hospitals' League."

2. Its objects should be :—

(*a*) The support of the voluntary hospitals within its sphere of operations.

(*b*) The provision of free maintenance in hospital for all members (and their dependents) who contribute to its funds in accordance with an agreed scale.

3. These objects should be carried out :—

(*a*) By the payment of the maintenance charge required by any voluntary hospital into which a member (or dependent) may be admitted for treatment.

(*b*) By the periodical distribution of all available funds, after the payment of necessary expenses, among the hospitals concerned, in proportion to the sum expended upon the treatment of members (or their dependents). Amounts paid for maintenance to be taken into account in this distribution.

This draft gives only the briefest outline of the suggested scheme, but it contains the germ of one which is in its principles fair to the contributors, and further it gives a just scale of contribution, it would become a handsomely paying proposition to all the hospitals concerned.

Yours faithfully,

W. Fenn, Chaplain.

General Infirmary, Salisbury.

---

Miss Mayer's "At Home," Imperial Nurses' Club, 137 Ebury Street, S.W.1. The Club's Birthday Week, during which both members and non-members will be entertained as guests every afternoon, is to begin this year with an "At Home" on Monday, November 27, from 8—10 p.m. A good programme of music has been arranged for each day, and an excellent concert for Sunday. It is hoped that as many members as possible will visit the Club with their friends during the week.

Messrs. Charles Zimmermann & Co. (Chemicals), Ltd., have sent us a specimen of Idozan, a form of iron, colloidal in character, in a neutral, permanent solution. In this form the iron (of which Idozan contains 5 per cent. Fe) is completely assimilated, without causing constipation, headache, or blackening of the teeth. It is generally acknowledged, though perhaps not so well appreciated, that iron must be given in large doses, but the unpleasant taste and often bulky nature of the usual iron compounds and the constipating effect are difficulties. With Idozan all these have been overcome. It is a labile colloidal solution free from acidity, containing 5 per cent. pure iron. It is pleasant to the palate, with no trace of "inkiness," does not stain the teeth in the least degree, and has *no* constipating action. It is completely soluble in both water and milk, and may also be given with tea, it being unaffected by the tannin usually present in this beverage.

The problem of maintaining the voluntary hospitals in Ulster has led a deputation of county hospital committeemen and others to wait on Sir Dawson Bates at the Home Office of the Northern Government. The removal of disabilities and the reform of the Poor Law were demanded, because of the old laws of 1765; pensions for hospital officers on the same scale as Poor Law and Civil Servants were asked for, and permission to the county councils to make grants also. Dr. Kidd and Dr. Thompson supported the proposals on behalf of the profession and the hospitals. Sir Dawson Bates gave a cautious but sympathetic reply, saying, however, that the larger question of hospital and Poor Law reform was difficult and required much care.

We have received from Messrs. Cadbury Bros., Ltd., a box of chocolates. As is well known, this firm's goods are produced from the best materials under ideal conditions by workers whose welfare is studied in every possible way. This handsome box is indeed a welcome Christmas present.

A Pound Day, Bazaar and Concert will be held on December 1, at the Royal Northern Hospital, Holloway Road, N.7. The bazaar opens at 12.30. Viscountess Ednam will be present to receive Pound Day Gifts at 2.30, and the concert, at which many well-known artistes have promised to appear, will begin at 6 p.m. An urgent appeal is made for generous contributions of gifts and money.

Two lectures on "Relative Values in Public Health," by Sir Arthur Newsholme, K.C.B., M.D., late Principal Medical Officer of the Local Government Board, will be delivered at Barnes Hall, Royal Society of Medicine, 1 Wimpole Street, W.1, on Thursdays, December 7 and 14, at 5.15 p.m. These lectures are open to the public. 1. Value of vital statistics, sanitary surveys and professional and popular education. Historical influence of general sanitation, specific sanitation and combined action. 2. Degrees of preventability of disease—increasing contribution of physiological hygiene. Fundamental work and conditions. Problem of money apportionment.

